# Transactions of the Physical Department of the Imperial Institute during the Year 1811

**Published:** 1812-05

**Authors:** 


*For the Medical and Physical Journal.*

TRANSACTIONS *of the* PHYSICAL DEPARTMENT *of the* IMPERIAL INSTITUTE *during the* YEAR 1811.

*Anatomy, Physiology, and Zoology.*

IN the account of the proceedings of the Institute last year, in noticing the researches upon the action of the nerves of the eighth pair in respiration, allusion was made to the important important experiments by which M. Legallois, physician at Paris, has proved that very young animals may live without respiring for a certain time, the duration of which is proportionate to their nearness to the period of birth; in other words, that the younger the animal is, the longer it is capable of living without breathing.

M. Legallois, having subjected very young animals to other lesions, has obtained results yet more singular, and has been enabled to resolve a question disputed by anatomists nearly two centuries; viz. the influence which the nerves have in the motions of the heart.

Having decapitated some of these animals, he observed that their heads continued to give signs of life, precisely the same time for each age that the animals of that age could pass without respiring; from which he concluded, that the heads died only from want of respiration.

It is further determined by the experiments of Fontana, that it is possible to prolong life in the decollated trunk by inflating the lungs. The immediate principal of the life of the trunk is then in the trunk itself.

But, on the other hand, it is known that the life of each part requires its direct communication with the spinal marrow by means of the nerves, and a free circulation of blood in the portion of medulla which supplies nerves to that part.

This being admitted, we should suppose that the simple destruction of a portion of spinal marrow ought only to affect those parts to which it gives nerves; but it happened otherwise in the experiments of M. Legallois. The destruc tion of a portion of marrow quickly killed the whole body, and consequently produced greater effect than decollation itself.

Upon attentively examining all the circumstances of this phenomenon, M. Legallois perceived that this lesion weakened and soon altogether stopped the circulation; the arteries emptied themselves. He therefore concluded, that in such cases death was produced by the motions of the heart being enfeebled.

This conjecture was verified by experiments. In diminishing, either by means of ligature on the arteries, or even by amputation, the number of parts to which the heart should supply blood, the power which remains in it is sufficient, because it has less efforts to make, and the lesion of the medulla is less quickly mortal. Thus an animal whose head is cut off, will die slower from a lesion of the spinal marrow, than if its head had remained on; and, as a partial lesion of the medulla, after some time, considerably diminishes the circulation in the parts to which the portion of marrow marrow destroyed gives nerves, the destruction of a portion of medulla gives the means of destroying, after a little time, another portion without causing death so speedily. Thus, when we have decapitated an animal, it is more easy to destroy its cervical marrow without killing the rest of its trunk; and, when we have destroyed its cervical marrow, it is easier to perform the same operation on the dorsal marrow; so that if we could supply them with the heart and lungs, we might make each of these amputated portions of the body live successively, without the others; and the chest which contains these organs can preserve its life a considerable time without the assistance of any of the other parts.

The general and direct result of these curious experiments is, that the motion of the heart depends entirely upon the spinal marrow which exercises its influence upon it, by means of the grand sympathetic. In this manner we can explain why the heart is affected by passions without immediately depending on the brain, and we are thus enabled to submit to the empire of the nerves the only muscular organ on which nervous influence was liable to some objections; in short, as the suppression of the brain does not immediately affect the motions of the heart, whilst that of the spinal marrow destroys them, the opinion advanced within some years by great physiologists, that the brain is not the only source of nervous action, but that each part of the nervous system also exercises a share in that action, is fully confirmed.

The count de Cessac, minister of war, and member of the class of the French language and literature, having consulted the class of sciences upon the means of preventing the ravages which certain worms make in the magazines of cloths and woollens, Messrs. de Lamarck, Vauquelin, Richard, and Bosc, have made a full report upon this important object.

These worms are the caterpillars of six or seven species of small night moths, which not only devour the hairs of animals, but also make of them small tubes, which serve them for clothing as well as a habitation. Many chemical agents will destroy these caterpillars, but most of them, if incautiously used, would do more mischief to the cloths than the insects themselves. However, heat may always be had recourse to, and in all cases it is well to prevent the multiplication of the caterpillars by destroying the moths, and using every means for preventing them getting into the magazines. The limits of this report do not admit of our detailing the particulars recommended by the commissaries for accomplishing these different ends.

Natural philosophers have long sought for the causes of the the phosphorescent appearance which the sea frequently exhibits. The late M. Peron published, some months before his death, a very complete work upon this curious phenomenon, in which he indicated a great number of animals that contributed to it, and which often vary in themselves, according to the climate in which the phenomenon manifests itself.

M. Suriroy, physician at Havre, excited by M. Peron, has examined the luminous animals of the port he inhabits, and has described one species globulous, about the size of a pin's head, and so numerous that it sometimes forms a thick crust on the surface of the water. Besides its spontaneous phosphorescence, it shines when it is irritated, and even when bruised.

M. Lamouroux, professor at Caen, has carefully examined certain small fish, known in Normandy by the name of *montée*, from their going in prodigious numbers up the rivers d'Orne, de Touque, and de Dive. They are commonly supposed to be the young of eels, but M. Lamouroux has found that they bear more resemblance to the conger, without, however, possessing all its characters; it is probable that they are the fry of a particular species, for it has been ascertained that several species of eels exist at the mouths of our rivers (in Normandy) not yet accurately described by naturalists.

*Medicine and Surgery.*

M. Chaussier, correspondent and professor in the faculty of medicine, has communicated a memoir upon that complaint so dangerous to lying-in women, known by the term *puerperal fever*, or *peritonitis*.

Physicians have long attributed it to a milky effusion, because they found, in the abdomen of persons who died of it, a serous fluid mixed with flakes resembling a caseous substance; but M. Chaussier has shewn that these substances have nothing in common with milk, except a false resemblance. He quotes instances of a similar disease attacking men and young girls; proves that it is of a catarrhal nature; explains, from the changes of constitution during pregnancy and parturition, why women in childbed are more exposed to it than other individuals; and, what is yet more important, declares that in many cases he has obtained the most marked success in puerperal fever, by the employment of vapour baths, and friction of mercurial ointments on the abdomen.

M. Itard, physician to the school of the deaf and dumb, has succeeded in restoring a case by perforating the tympanum, as practised in this country by Mr. A. P. Cooper.

 Amongst

Amongst the numerous operations to which the military surgeon is compelled by the casualties so frequent in battle, few are so hazardous, and so seldom attended with success, as amputation of the arm at the shoulder joint; and, of the many accidents that occur to interrupt the hope of the surgeon, none is more cruel than tetanos, or that convulsive rigidity which, in certain circumstances, seizes on the wounded, and hurries them to a death the more dreadful, because their intellectual faculties are not affected. M. le baron Larrey, whose experience in military surgery is proportioned to the murderous wars which have contributed to it, and to the various and remote theatres where he has been successively transported with the French armies, has presented to the class memoirs upon both these subjects. In the first he relates fourteen successful cases of amputation at the shoulder joint. In the second he states the almost miraculous effects which he has obtained from caustic *(du feu)* in tetanos, by applying it to those parts where he judged the centre of nervous irritation might be situated. Cold affusion, recommended by the English and German physicians, on the contrary, never afforded him any satisfactory results.

Another disease, which too frequently unites its ravages with those of war, is that kind of putrid fever which arises in places where men are crowded together in too great numbers, and which has been termed hospital-ship and jail fever. M. Masuyer, professor at Strasbourg, has addressed to the class a memoir, in which he affirms that acetite of ammoniac, or sp. mindereri, given in large doses, has produced the most marked effects, and considerably diminished the mortality in those hospitals in which the fever prevailed. Those at Paris are now so well regulated, that happily no opportunity of verifying the assertion of M. Masuyer has occurred; but the remedy has been tried in common putrid or adynamic fevers, and has been found to prevent the formation of the black crust or fur which covers the tongue and gums in such cases.

Among the medical works published this year by the class or its correspondents, we have chiefly to notice the treatise of M. Portal on the Nature and Treatment of Apoplexy; the second edition of the treatise on Organic Diseases of the Heart, by M. le baron Corvisart; the Medical Discourses, Memoirs, and Observations, of the late M. Desessarts; the great treatise on Hernia, by M. Scarpa, professor at Pavia; and the Manual of Practical Medicine, by M. Odier, professor at Geneva.

*Veterinary*

*Veterinary Art and Agriculture.*

It has long been known that the disease called *tourni* is caused by an animal in the class of intestinal worms, which exists in the brain of the sheep, and compresses or destroys that organ. Another disease in the same quadruped is occasioned by a worm called *douve*, which propagates in the biliary vessels of the liver; in short, some physicians suppose that the itch is owing to a small insect which is occasionally observed in the pustules produced in that complaint. M. Morel de Vinde, correspondent of the class, having remarked that a phthisis, which appeared on the suppression of an itch, had yielded to the internal use of sulphur, concluded that, being cured by the same means as the itch, it ought to depend on the same cause, that is, on the same parasitic animals which might have penetrated interiorly. He has extended this conjecture to several other diseases, and particularly to that named *pesoyne*, *pietain*, or *mal blanc*, which is an ulcer in the foot of the sheep. This malady, which, if neglected, quickly rots the foot and even the leg, and invariably destroys the animal, and for which no remedy has been found but the strongest caustics, has constantly been cured by a simple means devised by M. de Vindé, in consequence of the hypothesis which he had formed. His remedy consists in paring the horn of the foot till the white spot which the ulcer forms is seen, and in slightly rubbing the horn with a bunch of feathers dipped in aqua fortis. Some hours afterwards the sheep ceases to limp, and it is seldom necessary to repeat this simple operation. M. de Vindé has performed the experiment on more than fifty sheep without its ever failing, or making feverish and lose their milk, as frequently happen when other means have been pursued.

M. d'Audebert is engaged in a great work upon the relations which the diseases of animals have to those of man. M. Noyez, veterinarian at Mirepoix, addressed a memoir to the class upon the good effects which result from the shearing of domestic animals, such as the ox and the horse, both in the cure and prevention of certain diseases.

